# Outcomes of cochlear implants in patients with *PCDH15* mutations: a clinical study

**DOI:** 10.3389/fgene.2025.1541333

**Published:** 2025-05-22

**Authors:** Qingling Bi, Zhongyan Chen, Baoling Kang, Yong Lv, Yongyi Yuan, Yang Liu, Jianfeng Liu, Yuan Li

**Affiliations:** ^1^ Department of Otorhinolaryngology and Head and Neck Surgery, China-Japan Friendship Hospital, Beijing, China; ^2^ Beijing Angel Gene Medical Technology Co., Ltd., Beijing, China; ^3^ Key Lab of Hearing Impairment Science of Ministry of Education, Key Lab of Hearing Impairment Prevention and Treatment of Beijing, National Clinical Research Center for Otolaryngologic Diseases, College of Otolaryngology Head and Neck Surgery, Chinese PLA General Hospital, Chinese PLA Medical School, Beijing, China

**Keywords:** *PCDH15* gene, sensorineural hearing loss, genetic mutations, usher syndrome, cochlear implantation, auditory and speech outcomes

## Abstract

**Objectives:**

To explore molecular diagnoses in cochlear implantation (CI) recipients and evaluate CI outcomes in patients with *PCDH15* mutations.

**Methods:**

Whole-exome sequencing and biomedical informatics were used to identify potential genetic causes in 467 individuals with congenital sensorineural hearing loss. We reviewed six CI recipients with *PCDH15* mutations, assessing their CI outcomes and clinical features.

**Results:**

Nine *PCDH15* variants and a heterozygous variant in *CDH23* were identified in members of five families who underwent CI. Six of these were novel variants: exon 14–21 del, exon two del, exon 19 del, two splicing variants (c.2869-2A>C, c.1918-1G>A) in *PCDH15*, and c.209C>T in *CDH23*. All but one of the individuals with *PCDH15* mutations exhibited autosomal recessive inheritance; one showed both digenic and autosomal recessive inheritance. Variants in *PCDH15* contributed to Usher syndrome type 1F in patients 1 and 5, whereas the remaining four had isolated deafness (DFNB23). All six patients expressed satisfaction with their CI outcomes.

**Conclusion:**

CI significantly improved auditory and communication abilities in individuals with *PCDH15* mutations. Early intervention is critical for achieving favorable outcomes. Preoperative genetic testing in individuals with hearing loss provides valuable insights for predicting CI success, offering potential treatments for retinal degeneration in Usher syndrome and facilitating personalized genetic counseling.

## 1 Introduction

Deafness poses a significant threat to the quality of life of newborns in China, where approximately 2 in 1,000 children are born with hearing impairment (Collaborators, 2021). Genetic factors are responsible for 60% of congenital or early-onset deafness (Sloan-Heggen et al., 2016), and most cases involve severe to profound sensorineural hearing loss (SNHL), Unfortunately, effective treatments remain unavailable. For those with severe to profound SNHL, cochlear implantation (CI) offers their only hope. However, clinical observations indicate that 7%–14% of eligible patients experience suboptimal auditory improvement after CI, leading to a substantial waste of medical resources and depriving children of future treatment opportunities. The factors and mechanisms influencing CI outcomes remain unclear, although studies suggest a critical role for genetic background in determining CI success (Park et al., 2017). Advances in molecular genetics have enabled the identification of over 180 genes associated with syndromic and non-syndromic hearing loss (https://hereditaryhearingloss.org/). Genetic defects can lead to various pathological changes along the auditory pathway, potentially affecting CI outcomes. Investigations have sought to establish correlations between specific gene mutations and post-implantation results. It was concluded that defects of the spiral ganglion were correlated with poor CI performance while defects of the membranous labyrinth and hair cells were correlated with favorable CI performance (Eppsteiner et al., 2012). For example, mutations in *GJB2, SLC26A4, mtDNA12SRNA*, and *A1555G* are associated with favorable CI outcomes due to their intra-cochlear pathology (Dahl et al., 2003; Wu et al., 2008; Tono et al., 1998; Rouillon et al., 2006). In contrast, when the causative gene leads to lesions in the spiral ganglion or retro-cochlear auditory pathway, CI tends to be less effective (Eppsteiner et al., 2012). To address this critical clinical challenge, we aim to further evaluate the auditory-linguistic performance and neural pathway development abnormalities resulting from deafness-related genes. Therefore, we systematically extend our search for molecular genetics from patients affected by deafness. Among these, the impact of auditory intervention on patients with *PCDH15* gene mutation has drawn our attention.

In 1997, Wayne et al. discovered a novel USH1F locus (OMIM: 602083) for Usher syndrome (Wayne et al., 1996). In 2001, Alagramam et al. identified three clones of the human homologous *protocadherin-15* (*PCDH15*) gene, localized on chromosome 10q11.2-q21 (Alagramam et al., 2001). *PCDH15* belongs to the cadherin superfamily of calcium-dependent cell–cell adhesion molecules and is strongly expressed in the neurosensory epithelium of the cochlea, vestibular hair cells, retinal photoreceptors and brain (Ahmed et al., 2003). The *PCDH15* gene encodes a calcium-binding protein essential for connections between stereocilia in the inner ear. Mutant alleles of *PCDH15* are responsible for both Usher syndrome type 1F (USH1F) and autosomal recessive deafness (DFNB23). Patients with USH1F present with profound congenital deafness, vestibular areflexia, and pre-pubertal-onset retinitis pigmentosa (RP). Variants in *PCDH15* are responsible for 11%–19% of USH1 cases (Tsang et al., 2018). Several reports have documented the expression of *PCDH15* along with other Usher proteins, such as *cadherin-23* (*CDH23*) and *CLRN1*, at the synapses of hair cells and photoreceptors. *PCDH15* protein is believed to play an important role in synaptic maturation of the auditory pathway. Zallocchi et al. observed pre- and post-synaptic expression of *PCDH15* in inner hair cells (IHCs) and outer hair cells during synaptic development, as well as in mature IHCs (Zallocchi et al., 2012). Therefore, mutations in *PCDH15* can greatly affect auditory processing (https://www.proteinatlas.org/, accessed on 18 February 2024). Nishio et al. found that *PCDH15* gene expression was higher in the spiral ganglion than in other parts of the cochlea (Nishio et al., 2017), suggesting a poor CI outcome for *PCDH15*-positive patients. Histological study in the rat model revealed severe defects in cochlear hair cell stereocilia, collapse of the organ of Corti, and marked reduction of ganglion cells in adult *Pcdh15* (kci) mutants (Naoi et al., 2009).

A detailed review on navigating the Usher Syndrome genetic landscape by Micol B et al., indicate that patients with *PCDH15* mutations are significantly less likely to have a “Positive” outcome, as only 3/12 cases were reported to acquire good or excellent CI performance for this gene, underscoring a potential link between *PCDH15* gene and unfavorable outcomes (Liu et al., 2008; Brownstein et al., 2004; Wu et al., 2015; Grillet et al., 2009; Busi and Castiglione, 2024). These findings appear consistent with the results of a laser-microdissection-based gene expression study showing that *PCDH15* is highly expressed outside the cochlea (Nishio et al., 2017). Adverse CI outcomes in *PCDH15*-affected individuals may be due to pathology in SGNs or brainstem auditory nuclei, which are essential for CI functionality (Eppsteiner et al., 2012). Therefore, the CI performance substantially varies among patients with defective *PCDH15* (see Table 1) and can be suboptimal (Liu et al., 2008; Wu et al., 2015).

**TABLE 1:** Literature reports of CI outcomes in patients with *PCDH15* gene mutations.

Nation/year published	Proband	Age (Y)	Sex	Transcript	Nucleotide	Proteins	Mutation type	Age at CI	CI outcomes
Han Chinese (Taiwan, China)/2015 (Wu et al., 2015)	1		Male	uc010qht.2 NM_001354411	c.145G>T	p.E49X	het	2.2	Very Poor
				uc010qht.2 NM_001354411	c.4744delC	p.L1582fs	het		
	2		Male	NM_033056	c.1863_1864dup	p.S622fs	hom	1.9	Poor
	3		Male	NM_033056	c.4320_4328dup	p.P1441_P1443dup	het	4.7	Poor
				NM_033056	c.3451G>A	p.G1151R	het		
	4		Female	NM_033056	c.4812G>T	p.R1604S	hom	2.6	Very Poor
Ashkenazi/2004 (Brownstein et al., 2004)	5	7	Male	NM_033056	c.733C>T	p.R245X	hom	3y4m	Good
	6	4	Male	NM_033056	c.733C>T	p.R245X	hom	1y3m	Poor
	7	5	Male	NM_033056	c.733C>T	p.R245X	hom	1y10 m	Good
	8	4	Female	NM_033056	c.733C>T	p.R245X	hom	1y6m	Excellent
Shi Chinese/2021	1	8	Female	NM_033056	EX19-21del/c.1997 + 1G>A	-	het	9 m	Good
	2	33	Female	NM_033056	c.3441 del A/c.2528C>A	p.Lys1147 Asn/p.Ala843 Asp	het	13 y	Poor
	3	3	Male	NM_033056	c.733C>T/c.157+1G/A	p.Arg245*/-	het	11 m	Good
	4	4	Female	NM_033056	c.733C>T/c.2756del T	p.Arg245*/p.Mer919 Arg	het	10 m	Good
Li Chinese/2020	1	8	Male		c.4115delG/c.3490_3491insA	p.G1372Efs*4/p.M1164Nfs*12	het	2 y	Good
Nelson Chen/2022 (Chen et al., 2022)	1	21	Female	NM_001384140.1	c.60_61del	p.Phe21Ter	hom	early childhood	NA

Cochlear outcomes were classified using four arbitrary categories.

Category 1 (Excellent): Significant speech recognition improvement (75%–100% correct), high satisfaction, CAP 6–7.

Category 2 (Good): Noticeable speech recognition improvement (50%–74% correct), stable CAP 4–5.

Category 3 (Poor): Limited speech recognition improvement (25%–49% correct), dissatisfaction, CAP 2–3.

Category 4 (Very Poor): Minimal speech recognition improvement (<25% correct), major dissatisfaction, CAP 0–1.

To evaluate the effect of CI and enable the early detection of ocular and vestibular dysfunction in patients with *PCDH15* mutations, we combined electrophysiological assessments with routine clinical data to elucidate intrinsic relationships among genetic background, auditory neural pathway development, and auditory-linguistic outcomes. We aim to facilitate the prediction of CI outcomes through genetic testing.

We performed genetic analyses in six patients with *PCDH15* mutations from five families. In addition to clinical examinations, hearing and speech performance, vision, fundus, and balance were assessed after CI. This approach provides a foundation for gene therapy in treating deafness and highlights the importance of incorporating genetic prognostic factors into clinical decision-making, thereby optimizing surgical outcomes.

## 2 Patients and methods

### 2.1 Patients and clinical assessments

For exome sequencing analysis, 467 individuals with congenital, profound sensorineural hearing loss were recruited. Detailed medical and family histories were collected for all patients. In individuals with *PCDH15* mutations who underwent CI, the following assessments were performed: high-resolution temporal bone computed tomography (CT) and magnetic resonance imaging (MRI) before implantation; behavioral audiometry; electrically evoked auditory brainstem response (EABR); monosyllable and bi-syllable word recognition; short sentence recognition; Categories of Auditory Performance (CAP); and Speech Intelligibility Rating (SIR) to evaluate auditory and speech outcomes.

Materials [Speech and Language Rehabilitation Wordlist for Hearing-Impaired Children, developed by Sun et al. (Xibin et al., 2009)] present in quiet were utilized to evaluate speech perception performance. Stimuli were presented in the sound field at 65 dBA via a single loudspeaker; subjects were seated directly facing the loudspeaker at a 1 m distance. Vestibular and balance function were assessed using vestibular evoked myogenic potentials (VEMP) and the sensory organization test (SOT). Visual fields and retinal function were evaluated using optical coherence tomography (OCT), full-field electroretinography (ffERG), and ERG with DTL electrodes.

This study was approved by the China–Japan Friendship Hospital Ethics Committee [approval number (2023) KY no. 217]. Informed consent was obtained from the parents of minors under the age of 18.

### 2.2 Exome sequencing and bioinformatic analyses

Blood samples from 467 individuals with congenital profound hearing loss and their family members were collected for DNA extraction. Exome-enriched genomic libraries were prepared using the Agilent SureSelect Human Expanded All Exon V6 kit and sequenced on an Illumina NovaSeq6000 platform with an average coverage of 100×. After sequencing the target regions, quality control was conducted using Trim Galore. Clean data were obtained by filtering out adapter sequences and low-quality reads. The Burrows-Wheeler algorithm was used to align clean sequences to the human reference genome (hg19 for nuclear genes or GRCh37 for mitochondrial genes). Variants, including SNPs and Indels, were detected using GATK. ANNOVAR was utilized to obtain information regarding the variants, such as minor allele frequency (MAF), variant consequences, altered protein function, gene associations, and related diseases. Changes in protein function were predicted using the VarSome, MutationTaster, PolyPhen-2, and SIFT databases. Variants with MAF >0.001, based on gnomAD or an in-house Chinese Exome Database, were excluded as previously described (Li et al., 2021). Confirmatory Sanger sequencing with specific primers was performed for selected variants. Based on the wild-type three-dimensional model of the *PCDH15* protein (https://swissmodel.expasy.org/), potential effects of the identified variants on *PCDH15* protein function were predicted using SPDBV 4.10 software (https://swiss-pdb-viewer.software.informer.com/4.1/) and PyMOL (https://www.pymol.org/). Copy number variation (CNV) analysis was conducted by aligning short-read fragments generated by NGS analysis to the reference genome, determining their positions, generating BAM files, and visualizing the data using IGV software. Regions where copy number variation occurred showed significant differences in sequencing depth compared with normal regions, allowing the estimation of CNV based on the depth of sequencing across gene regions.

## 3 Results

### 3.1 Clinical findings in 5 PCDH15-affected families

Hearing threshold tests using pure tone or behavioral audiometry revealed bilateral severe to profound sensorineural hearing loss in all *PCDH15*-affected probands. Pre-implantation cranial MRI and high-resolution temporal bone CT (HRCT) indicated normal ear structures and brain development in all *PCDH15*-affected individuals. Six patients from five families underwent CI and formal auditory-oral rehabilitation. The post-implantation follow-up period ranged from 5 months to 10 years. CI-aided hearing threshold levels [Rt/Lt (dB)] in the probands ranged between 20 and 55. The auditory and speech assessments post-implantation are detailed in Table 3. No history of genetic disorders was identified in these five families.

**FIGURE 1:**
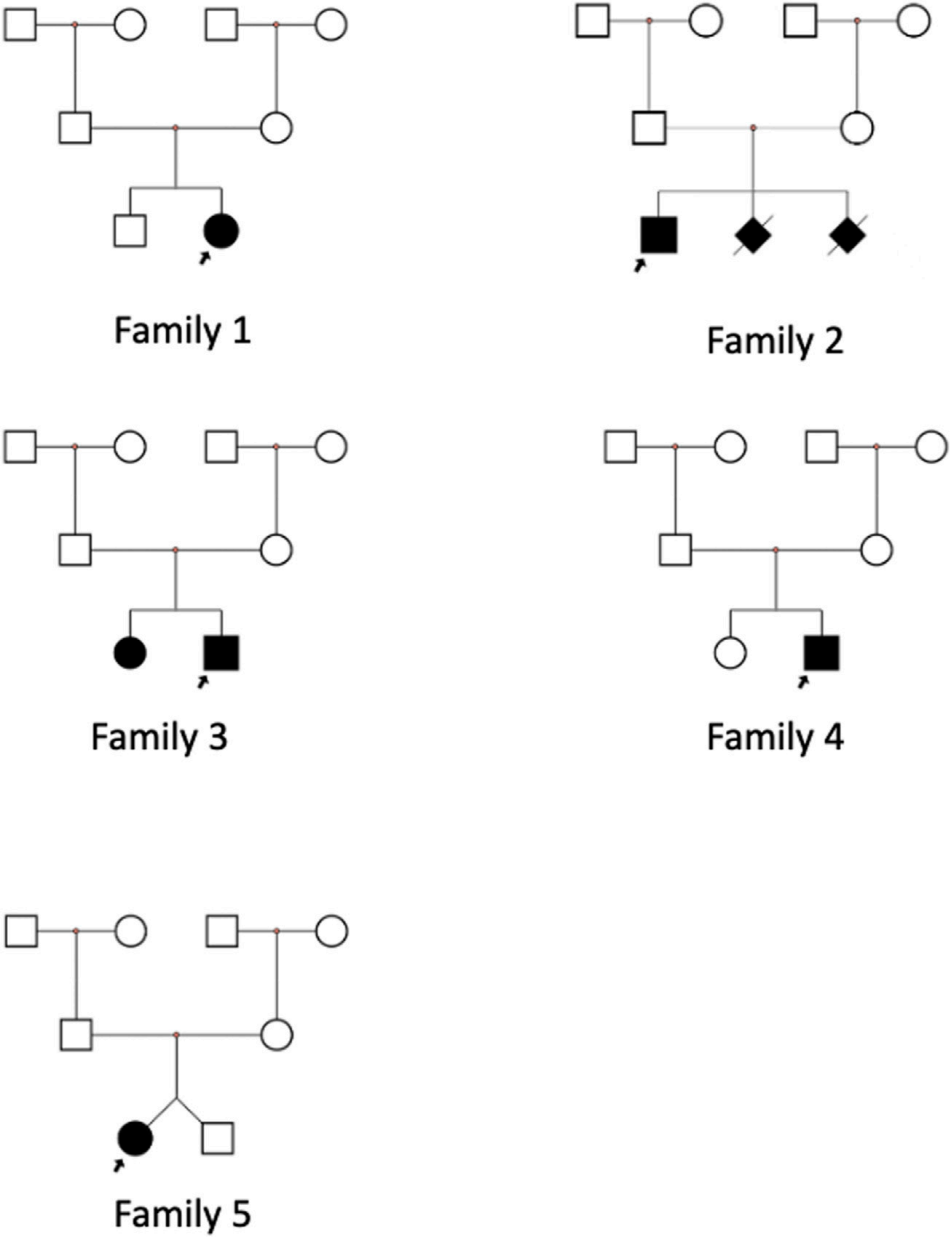
Pedigrees of the *PCDH15*-affected families.

**FIGURE 2:**
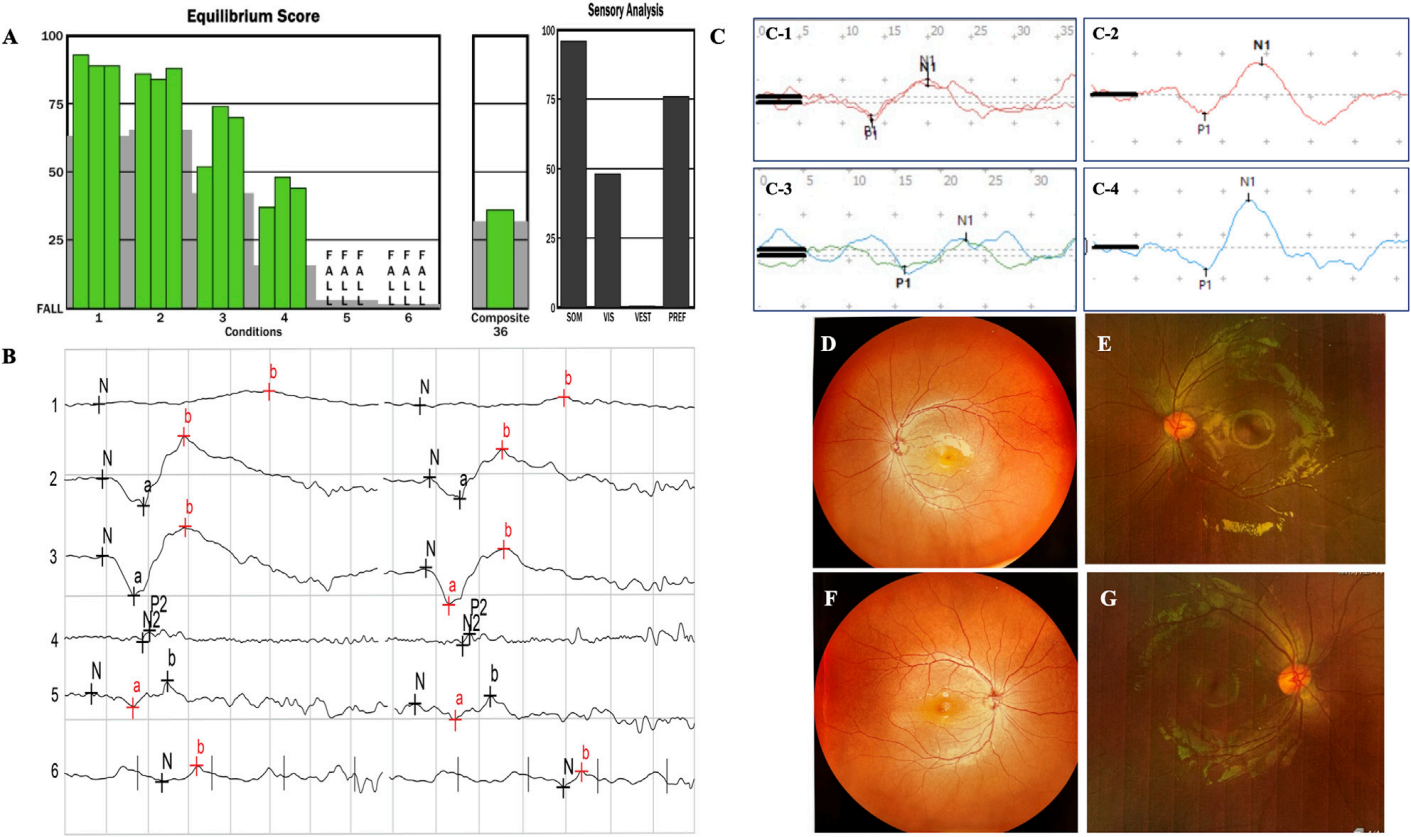
In family 1, the proband is a 4-year-old girl with a healthy brother. She developed typical USH1F at age 2. The fundus photographs of the retina are normal **(D,F)**, with uncorrected visual acuities of OD 0.2 and OS 0.32. The ERG results indicated abnormalities in cone cells, rod cells, and associated bipolar cells **(B)**. The patient fell during SOT tests 5 and 6 **(A)**; no stable reproducible waveforms were evoked during oVEMP and cVEMP tests, suggesting abnormal vestibular function **(C-1,C-3)**. **(E,G)**, **(C-2,C-4)** from family 2, with recessive non-syndromic hearing loss (DFNB23), showed good evoked VEMP and normal fundus images.

**FIGURE 3:**
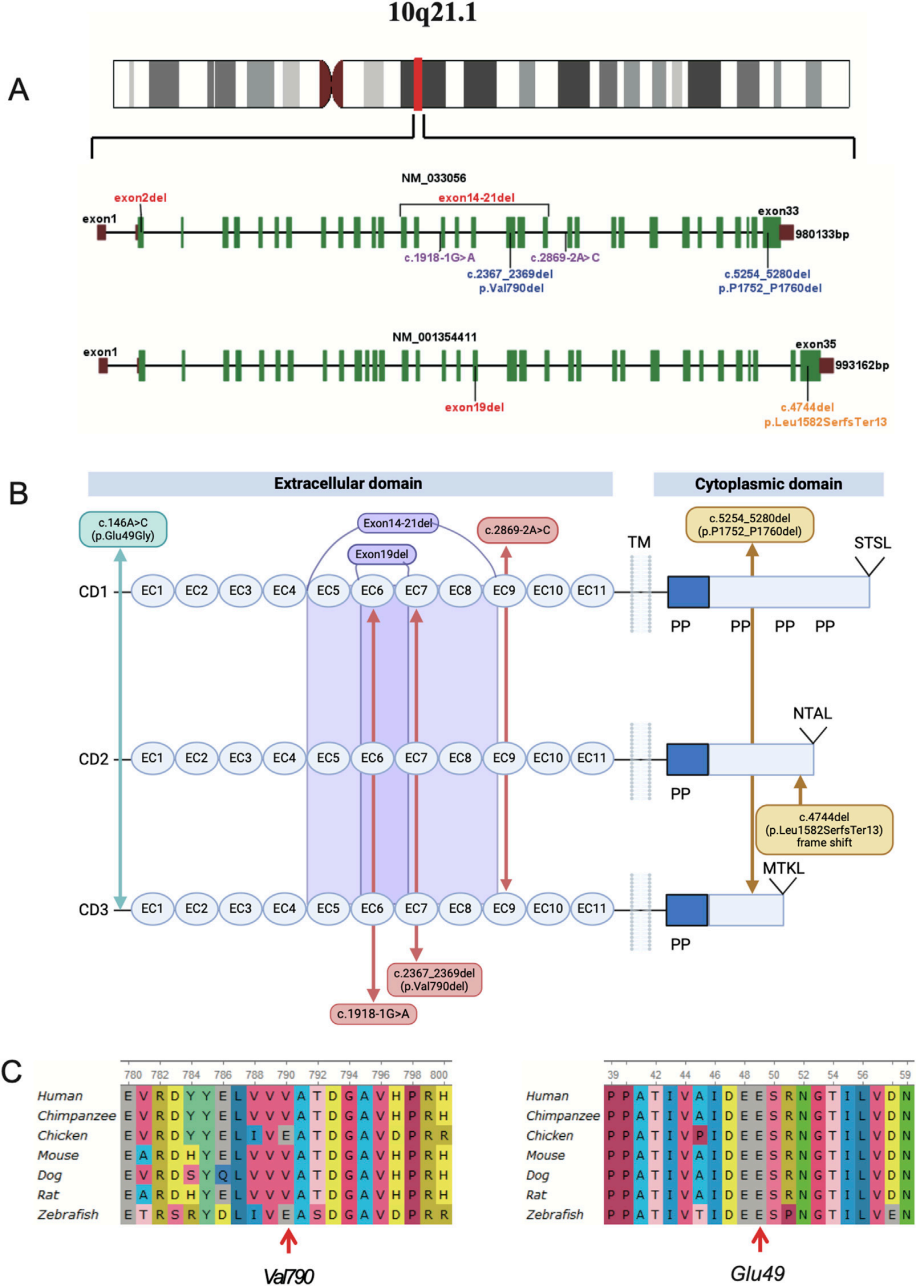
**(A)**, chromosomal location and gene structure map of the *PCDH15* gene. The *PCDH15* gene spans a genomic region of 980 kb, composed of 33 exons (NM_033056), or 993 kb, composed of 35 exons (NM_001354411). **(B)**, eight different variants identified in the six affected individuals are mapped to the *PCDH15* protein. **(C)**, the two variants are evolutionarily conserved as shown in multiple sequence alignment containing seven different species from *Xenopus* to *Homo sapiens*.

In family 1, the proband was a 4-year-old girl with a healthy brother (Figure 1, family 1). She developed typical USH1F at age 2. Fundus photography of the retina was normal, with uncorrected visual acuities of OD 0.2 and OS 0.32 (Figures 2D,F). ERG findings indicated abnormalities in cone cells, rod cells, and associated bipolar cells (Figure 2B). She fell during SOT tests 5 and 6; no stable reproducible waveforms were evoked during oVEMP and cVEMP tests, suggesting abnormal vestibular function (Figures 2A,C-1,C-3). She underwent sequential CI at an early age and achieved excellent outcomes.

In family 2, the proband was a 7-year-old boy with recessive non-syndromic hearing loss (DFNB23) (Figure 1, family 2; Figures 2C-2,C-4,E,G). He achieved excellent speech outcomes after sequential CI at the ages of 2 and 4 years.

In family 3, the proband was a 3-year-old boy, the second child of healthy, non-consanguineous parents. His older sister exhibited the same symptoms (Figure 1, family 3). Both siblings were diagnosed with isolated deafness (DFNB23). They underwent unilateral CI and achieved satisfactory outcomes.

In family 4, the proband was a 17-month-old girl with severe hearing loss and vestibular dysfunction (Figure 1, family 4). Her parents declined fundoscopy and ERG. She underwent simultaneous bilateral CI at 1 year of age and has since shown consistent responses to sound. Speech audiometry is not yet available due to the short follow-up period after CI.

In family 5, one of two twin sisters was diagnosed with isolated profound deafness (Figure 1, family 5). She underwent CI at 19 months, with excellent results.

### 3.2 PCDH15 variants identified in 6 patients from 5 families

The *PCDH15* gene spans a 980 kb genomic region composed of 33 exons (1955 amino acids, GenBank acc. no. NP_149045.3). The *PCDH15* protein consists of 11 extracellular cadherin repeats (EC), a transmembrane region (TM), and a cytoplasmic domain that includes poly-proline repeats (PP) and unique C-terminal PDZ-binding interfaces. Hair cells express three main *PCDH15* isoforms (CD1, CD2, and CD3), which differ in their cytoplasmic domains (Figures 3A, B) (Alagramam et al., 2001; Wu et al., 2015; Grillet et al., 2009; Busi and Castiglione, 2024).

This study identified nine variants of the *PCDH15* gene (NM_033056) and one missense mutation in the *CDH23* gene (NM_022124.6) through exome sequencing (Table 3; Figure 3), which were confirmed by Sanger sequencing (Supplementary Figure S1). Among these mutations, none of the CNVs, two splicing variants in *PCDH15*, and a missense mutation in *CDH23* had been previously reported or curated in the Human Gene Mutation Database (HGMD). These unreported mutations include the splicing mutation c.2869-2A>C and the exon 14–21 deletion in family 1; c.1918-1G>A in *PCDH15* and c.209C>T (p.Ser70Phe) in *CDH23* in family 2; the exon 19 deletion in family 3; and the exon 2 deletion in family 4. VarSome ACMG analysis predicted that most of these variants were pathogenic or likely pathogenic (Table 2), whereas two non-frameshift variants (c.2367_2369del and c.5254_5280del) and the missense variant c.146A>C were classified as variants of uncertain significance.

**TABLE 2:** Clinical features of patients with *PCDH15* gene mutations.

Patient	Age	Sex	Phenotype	Age at CI (months)	Side	Aided CI hearing threshold (dB HL)				Device/Processor	Monosyllable recognition (closed-set) (%)		Word recognition (closed-set) (%)	Short sentence recognition (open-set) (%)	CAP	SIR	Follow-up duration
						0.5 kHz	1 kHz	2 kHz	4 kHz		Vowel	Consonant					
1	4Y4m	F	Usher IF	2021.3.15 (L) 2023.7.13 (R)	Sequential bilateral	(L)30 (R)45	25 35	35 35	30 35	L: Conserto/Rondo 2 R: Synchrony/Rondo 2	88%	84%	100%	100%	7	3	41 m
2	7Y4m	M	DFNB23	2019.3 (L) 2021.12 (R)	Sequential bilateral	(L)40 (R)45	40 35	35 30	35 35	L: Conserto/Rondo 2 R: Synchrony/Rondo 2	92%	100%	100%	100%	8	5	65 m
3	2Y10 m	M	DFNB23	2023.2.10	Right	35	25	20	20	Nucleus522/N6	92%	76%	100%	100%	6	4	18 m
4	12Y5m	F	DFNB23	2014.1	Right	40	30	25	25	Nucleus422/	100%	100%	100%	100%	9	5	125 m
5	1Y5m	M	USH1?	2024.3.27	Simultaneous bilateral	(L)35 (R)40	45 45	40 55	45 50	B: Synchrony/Rondo 2	NA	NA	NA	NA	2	NA^a^	5 m
6	4Y4m	F	DFNB23	2022.2	Left	40	30	35	35	Nucleus522/N6	88%	88%	100%	100%	7	4	30 m

^a^ Patient 5 recognizes “a” and “i” using the Ling Six sound check.

CAP, categories of auditory performance; SIR, speech intelligibility rating; NA, data not available or not useful for statistical analysis.

In family 1, genetic analysis of patient 1 confirmed a splicing variant (c.2869-2A>C) and a large deletion (involving exons 14–21) in the *PCDH15* gene, inherited from her mother and father, respectively, consistent with autosomal recessive inheritance. Sanger sequencing revealed that the proband was heterozygous for the splicing site (Supplementary Figure S1). The SNV c.2869-2A>C affects an acceptor splice site in intron 21 of the *PCDH15* gene. Variants influencing the donor or acceptor splice site at the ninth EC repeat (EC9, amino acids 948–1,034) typically lead to a loss of protein function (https://rddc.tsinghua-gd.org/) (Baralle and Baralle, 2005). The deletion of exons 14 to 21 results in the loss of 426 amino acids, leading to protein truncation and loss-of-function variants in *PCDH15* that are known to be pathogenic (Alagramam et al., 2001; Ahmed et al., 2003; Ahmed et al., 2001). Both mutations were classified as likely pathogenic according to ACMG criteria.

In family 2, heterozygous mutations c.2367_2369del and c.1918-1G>A in the *PCDH15* gene were identified through NGS analysis and confirmed by Sanger sequencing in patient 2 (Supplementary Figure S1 Family 2). The first mutation (c.2367_2369del) was present in the father; the second mutation (c.1918-1G>A) was present in the mother. The novel splicing variant c.1918-1G>A, located in exon 16, is predicted to disrupt RNA splicing in the EC6 domain (amino acids 638–715), whereas the non-frameshift mutation c.2367_2369del has been previously reported (Zhan et al., 2015; Zheng et al., 2020). Moreover, the proband carried a heterozygous c.209C>T (p.Ser70Phe) mutation in *CDH23*, suggesting digenic inheritance involving mutations in both *CDH23* and *PCDH15*. According to ACMG guidelines, c.1918-1G>A was classified as likely pathogenic, whereas c.2367_2369del and c.209C>T were variants of uncertain significance.

In family 3, the proband (Figure 3) is of Man Chinese descent. Whole-exome sequencing showed that the proband carried a compound heterozygous variant in the *PCDH15* gene. A heterozygous frameshift mutation (c.4744del, p.Leu1582SerfsTer13) was inherited from his father. This mutation has been previously reported as segregating with USH1 in one family (Wu et al., 2015). The second mutation was a small deletion involving exon 19, inherited from his mother. The proband’s affected older sister (patient 4) carried the same two mutations. The frameshift variant leads to a premature stop codon at position 1,582, occurring in the last exon of the transcript, which is likely to escape nonsense-mediated decay (NMD) and result in a truncated protein. According to ACMG guidelines, both variants were predicted to be likely pathogenic.

In family 4, the proband (Figure 1, family 4) is the second child of non-consanguineous parents. He has a healthy sister. Whole-exome sequencing confirmed a homozygous deletion of exon 2 in the proband (Figure 3). This ΔEx2 deletion was heterozygous in both parents, and the proband’s sibling was confirmed to be a carrier. This CNV has not been previously reported. The deletion affects translation initiation codons, leading to a frameshift and a premature stop codon. According to ACMG guidelines, the CNV was classified as likely pathogenic.

In family 5, the proband was a girl with a healthy twin brother. The girl had impaired hearing. Trio whole-exome sequencing revealed that the proband carried a compound heterozygous mutation in the *PCDH15* gene. The first mutation, a non-frameshift deletion (c.5254_5280del, p.Pro1752_Pro1760del), was inherited from her father and has been previously reported in association with neurodevelopmental disorders (Gao et al., 2019). The second mutation, a missense mutation (c.146A>G, p.Glu49Gly), was inherited from her mother. Both variants were classified as of uncertain significance according to ACMG guidelines.

To examine the evolutionary conservation of the two mutated amino acids in patient 2 (c.2367_2369del, p.Val790del) and patient 6 (c.146A>G, p.Glu49Gly), *PCDH15* protein sequences from seven species, ranging from *Xenopus* to *Homo sapiens*, were retrieved from reference sequences on UniProt (https://www.uniprot.org/) for multiple sequence alignment using UGENE (http://ugene.net/). As shown in Figure 3C, the Val790 and Glu49 residues in the affected individuals are evolutionarily conserved. MUpro (http://mupro.proteomics.ics.uci.edu/) was utilized to predict the impact of the mutant and wild-type amino acids on protein folding free energy. This prediction indicated that p.Glu49Gly led to a decrease in protein stability (ΔG = −1.8909115). The mutant residue (Gly49) is smaller than the wild-type residue (Glu49), and the wild-type residue carries a negative charge, whereas the mutant residue is electrically neutral. The HOPE online server (https://www3.cmbi.umcn.nl/hope/) predicted that the wild-type residue participates in metal-ion [Ca (Sloan-Heggen et al., 2016)+] coordination. Upon mutation, the loss of the negative charge in glutamic acid destabilizes the interaction with calcium (Ca), which may impair the stability of the protein’s structural domain (Supplementary Figure S2). Several *in silico* tools were applied to predict the pathogenicity of the detected *PCDH15* mutations (Supplementary Table1). It is well-known that loss-of-function variants in *PCDH15* (ClinGen: Definitive) are known to be pathogenic (Alagramam et al., 2001; Ahmed et al., 2003; Ahmed et al., 2001). So all variants in the study were predicted to be deleterious.

## 4 Discussion

*PCDH15* is a member of the cadherin superfamily of calcium-dependent adhesion glycoproteins, highly expressed in the neurosensory epithelium of the cochlea, vestibular hair cells, and retinal photoreceptors. *PCDH15* and CDH23 are components of kinociliary links, transient lateral links, and tip links (Siemens et al., 2002; Lagziel et al., 2005; Ahmed et al., 2006; Senften et al., 2006; Kazmierczak et al., 2007; Rzadzinska et al., 2005). While Protocadherin-15 is an essential component of tip-links in hair cells. Research suggests that CDH23 interacts with *PCDH15* to form specialized tip links that gate the mechanoelectrical transduction (MET) channels in hair cells (Grillet et al., 2009), in addition to the well documented role for *PCDH15* proteins in stereocilia development, *PCDH15* protein likely function in synaptic maturation as well and is necessary for morphogenesis and polarity of the hair bundle. Variants in the *PCDH15* gene can disrupt stereociliary tips and interactions with transmembrane proteins (Tmc1,Tmhs and Tmie) which are essential for targeting *PCDH15* protein to plasma membrane and for MET, leading to functional impairment of the MET machinery (Pepermans and Petit, 2015). It may also compromise the synaptic connection between hair cells and spiral ganglion neurons (SGN), thereby impairing the transmission of auditory signals (Ahmed et al., 2006; Kazmierczak et al., 2007). Mutant alleles in *PCDH15* may contribute to isolated forms of profound deafness (DFNB23, phenotype ID: 609533) and Usher syndrome subtype USH1F, characterized by congenital profound sensorineural hearing loss, progressive RP, and vestibular impairment.

At least 211 mutations in *PCDH15* have been curated in the HGMD. The prevalent founder variant c.733C>T (p.Arg 245Ter) in *PCDH15* is unique to Ashkenazi Jews, with an occurrence rate of 50%–60% (Ben-Yosef et al., 2003). Pathogenic mutations are distributed throughout the *PCDH15* gene, without any apparent hot spots. The mutation spectrum in our data suggests that the nine mutations identified are dispersed, consistent with previous findings. In six *PCDH15*-affected individuals from five families, we identified 10 variants in *PCDH15* and *CDH23* (Table 3), six of which had not been previously reported: all the CNVs, two splicing variants in *PCDH15*, and a mutant allele c.209C>T in *CDH23*. The identification of these novel mutations broadens the spectrum of genetic deafness. Nearly all the mutations described are predicted to result in premature termination of the protocadherin protein. Several deletions were also identified. The *PCDH15* gene spans nearly 1 Mb, with a corresponding 7,021 bp open reading frame (ORF), and the first three exons cover 0.42 Mb. The high rate of deletions is likely a consequence of this unusual gene structure. Patient 1 had compound mutations, including a large copy-number-loss variant spanning exons 14 to 21 and a novel splice variant. The CNV deletion is estimated to be 157 kb, resulting in the loss of 426 amino acids. The c.2869-2A>C variant is expected to disrupt RNA splicing within intron 21, leading to truncation of *PCDH15* in the extracellular domains (EC5-EC9) (Baralle and Baralle, 2005). The literature suggests that this broad deletion contributes to the development of Usher syndrome (Vache et al., 2020). The patient developed USH1F at the age of 2 years. Patient 5 had homozygous deletions in exon 2, which is the critical first coding region for *PCDH15* protein translation. The deletion is estimated to be 119 bp in size, altering the predicted translation-initiation codons and resulting in aberrant protein translation, leading to synthesis of truncated proteins. There is evidence that the presence of the first few exons is essential for adhesion and impacts the mechanical properties of tip links (Kazmierczak et al., 2007). The child, at 18 months of age, has not yet achieved normal ambulation and tends to fall frequently. Clinically, USH1 is strongly suspected. Due to the patient’s young age and the family’s limited understanding of Usher syndrome, an ophthalmologic evaluation under anesthesia cannot be conducted at this time. However, long-term follow-up should include funduscopic examination and ERG to detect pre-symptomatic RP.

**TABLE 3:** Phenotypes of patients with *PCDH15* mutations.

Family/Patient	Gene	Chromosome position	Transcript	Nucleotide	Classification	Mode of inheritance	Mutation type	Inherited from	Isoforms	ACMG/AMP variant classification
1	PCDH15	chr10:55721654	NM_033056	c.2869–2A>C	Splicing	AR	het	Mother	CD1, CD2, CD3	Likely pathogenic
			NM_033056	Exon14-21del	CNV (deletion)		het	Father		Likely pathogenic
2	PCDH15	chr10:55782809–55782811	NM_033056	c.2367_2369del (p.Val790del)	Non-frameshift (deletion)	AR, DR	het	Father	CD1, CD2, CD3	Uncertain significance
		chr10:55849824	NM_033056	c.1918-1G>A	Splicing		het	Mother		Likely pathogenic
	CDH23	chr10:73269902	NM_022124	c.209C>T (p.Ser70Phe)	Missense		het	Father		Uncertain significance
3,4	PCDH15	chr10:55569060	NM_001354411	c.4744del (p.Leu1582SerfsTer13)	Frame shift	AR	het	Father	CD2	Likely pathogenic
			NM_001354411	Exon19del	CNV (deletion)		het	Mother	CD1, CD2, CD3	Likely pathogenic
5	PCDH15	chr10:56423902–56424091	NM_033056	Exon2del	CNV (deletion)	AR	hom	Father and mother	CD1, CD2, CD3	Likely pathogenic
6	PCDH15	chr10:55582205–55582232	NM_033056	c.5254_5280del (p.P1752_P1760del)	Non-frameshift	AR	het	Father	CD1, CD2, CD3	Uncertain significance
		chr10:56287583	NM_033056	c.146A>C (p.Glu49Gly)	Missense		het	Mother	CD1, CD2, CD3	Uncertain significance

Sequencing of the entire coding region of *PCDH15* revealed that patient 2 carried compound heterozygous mutations (c.2367_2369del and c.1918-1G>A) in *PCDH15* and a heterozygous missense mutation (c.209C>T) in *CDH23*, reflecting both autosomal recessive and digenic traits. The c.1918-1G>A mutation alters a splicing site and is predicted to cause exon 16 skipping, leading to a truncated protein. However, the significance of the novel *CDH23* variant is unknown. CDH23 interacts with *PCDH15* in the tip links of inner hair cells, and digenic interactions have been observed in both mice and humans, suggesting possible modification among genes associated with Usher syndrome. Digenic inheritance was suggested as an explanation for the cause of USH1 (Zheng et al., 2005). However, Ahmed et al. argued that the evidence supporting this hypothesis was not robust (Ahmed et al., 2008). Currently, patient 2 is 7 years old and exhibits no signs of visual disability or balance disorders, mimicking non-syndromic hearing loss. However, he is too young for a conclusive diagnosis. Most USH1 patients typically develop RP near puberty or even in the second or third decade of life. Therefore, close long-term follow-up is imperative. His mother subsequently had two pregnancies, and genetic analysis of amniotic fluid revealed that both fetuses carried the same compound heterozygous mutations in the *PCDH15* gene. Consequently, the family decided to terminate both pregnancies. To enhance the likelihood of conceiving a child with optimal auditory capabilities, we recommend using third-generation *in vitro* fertilization (IVF) techniques combined with preimplantation genetic testing (PGT) to ensure the birth of a healthy child without hearing impairment. This case shows that an individual’s genetic profile can provide mechanistic insights while supporting personalized treatment planning, tailored genetic counseling, and prenatal diagnosis.

In family 3, the proband carried a c.4744del (p.Leu1582SerfsTer13) frameshift mutation and a deletion in exon 19. His sister exhibited the same mutations. The deletion of exon 19 in *PCDH15* suggests splice variants with fewer cadherin repeats in the predicted calcium-binding domains (partial EC6 and EC7). The c.4744del frameshift mutation leads to the premature introduction of a stop codon, resulting in truncated proteins. The c.4744del mutation is predicted to affect only the *PCDH15-CD2* isoform. There are three common human *PCDH15* isoforms, which differ in their cytoplasmic domains (*PCDH15-CD1, PCDH15-CD2,* and *PCDH15-CD3*); each isoform has a specific role in the sensory hair bundle. In immature auditory hair cells, the various *PCDH1*5 isoforms (CD1, CD2, and CD3) are interchangeable for tip-link formation, whereas PCDH15-CD2 is essential in mature auditory hair cells (Pepermans and Petit, 2015). The absence of *PCDH15-CD2* leads to non-syndromic profound deafness in humans (Pepermans et al., 2014). Mice lacking pcdh15-CD1 and pcdh15-CD3 are capable of forming normal hair bundles and tip links; they also maintain hearing. However, pcdh15-CD2-deficient mice are deaf, with abnormally polarized hair bundles and no kinociliary links, although vestibular function remains intact (Webb et al., 2011; Le Guedard et al., 2007). A similar mechanism is likely involved in our case; our clinical observations are consistent with the pathogenic role observed in a pcdh15*-CD2* knockout mouse model. The deaf siblings (patients 3 and 4) are characterized by a recessive form of deafness, DFNB23, with preserved peripheral vestibular function. Patient 6 has compound heterozygous variants, including c.5254_5280del and c.146A>G. The c.5254_5280del non-frameshift mutation in exon 30 results in the loss of nine amino acids, which is predicted to alter the proteins within the cytoplasmic region. The c.146A>G transversion predicts an amino acid substitution of glutamate with glycine at codon 49; a previously reported mutation in this region was linked to autosomal recessive non-syndromic deafness. This mutation is near EC1, a region critical for adhesion. HOPE server predictions highlighted a critical role of Glu49 in calcium ion (Ca^2+^) coordination. The loss of the negatively charged carboxyl group in the mutant (Gly49) likely destabilizes Ca^2+^ binding, potentially compromising the structural integrity of the EC1 domain essential for *PCDH15*-mediated adhesion. DFNB23 is partly caused by defects in adhesion. The patient exhibited isolated deafness without vestibular or retinal impairment, suggesting a strong genetic correlation with the clinical features. Functional null alleles in *PCDH15* typically cause USH1, whereas missense mutations in conserved motifs or non-frameshift mutations that alter one or more amino acids, retaining some residual protein function, are commonly associated with recessive deafness.

In our series, six affected patients underwent successful CI before the age of 3. Patient 5 underwent CI only 5 months prior to this report. The child has shown normal latency and well-defined EABR waveforms III and V, with good postoperative auditory behavior. However, speech recognition depends on the development of hearing skills and the maturation of the auditory pathways. As she continues auditory speech development, she remains in speech rehabilitation training and is currently able to discriminate between “a” and “i” from the Ling Six sound check. Therefore, speech audiometry is not yet appropriate. Based on audiological and electrophysiological assessments, as well as clinical observations, no evidence of retro-cochlear lesions was found in our five Chinese families. More cases need to be analyzed to further explore these possibilities.

The poor CI outcomes in patients with *PCDH15* mutations documented in the literature may be attributed to several factors, including the absence of previous exposure to auditory stimulation and language use for those implanted at older ages, lack of engagement in structured auditory-verbal rehabilitation, or discontinuation of device use. Although genetic etiology plays a critical role in CI outcomes, it is not the sole determinant. Many other factors influence outcomes, including age at implantation, duration of deafness, inner ear anatomy, surgical technique, device characteristics, rehabilitation training, and family support. It is essential to emphasize the importance of individualized patient care.

To improve outcomes for patients with *PCDH15* mutations, early diagnosis is increasingly recognized as a pivotal step toward timely treatment, which can significantly impact disease management and results. *PCDH15* mutant alleles can cause either USH1F or non-syndromic hearing loss. Timely CI can significantly enhance auditory function and improve communication skills. Early diagnosis of USH1F should prompt anticipatory interventions to address the progressive loss of vision, such as recommending light avoidance and vitamin A supplementation. There are ongoing efforts to use gene replacement and editing, antisense oligonucleotides, and small-molecule drugs to halt RP progression and restore vision; these approaches hold promise for improving patients’ quality of life.

### 4.1 Limitations

While this study provides valuable clinical insights into the association between PCDH15 mutations and cochlear implant outcomes, its focus on phenotypic correlations inherently limits mechanistic depth. Future work integrating structural biology and functional genomics will clarify potential mechanisms, ultimately informing targeted therapies such as gene-editing or small-molecule modulators to rescue PCDH15 function.

In summary, our findings support the use of CI in patients with *PCDH15* mutations. Early CI is an effective intervention with favorable outcomes.

## Data Availability

The original contributions presented in the study are publicly available. This data can be found here: NCBI databases, accession numbers SUB15335605 and SUB15332707.
