# Supplementary material for: Outcomes of cochlear implants in patients with PCDH15 mutations: a clinical study
Source: Front Genet. 2025 May 22;16:1541333. doi: 10.3389/fgene.2025.1541333 (PMC12141853; doi:10.3389/fgene.2025.1541333)
Supplement: Supplementary file 1 [file Table1.docx]

*Supplementary Table: In silico* assessment of variants pathogenicity

| **Variants** | **SIFT** | **Mutation Taster** | **PROVEAN** | **CADD** | **MutPred2** | **RDDC** |
| --- | --- | --- | --- | --- | --- | --- |
| c.2869-2A>C | - | Deleterious | - | 34 | - | 0.5947 |
| c.2367_2369del | - | Deleterious | Deleterious(-7.942) | - | 0.70902 | 0.1712 |
| c.1918-1G>A | - | Deleterious | - | 34 | - | 0.6321 |
| c.209C>T(p.Ser70Phe) | Damaging(0.034) | Polymorphism | Neutral(-1.54) | 23.4 | 0.429 | 0.0115 |
| c.4744del | - | Deleterious | - | - | 0.33518 | 0.4669 |
| c.5254_5280del  (p.Pro1752_Pro1760del) | - | Deleterious | Neutral(-0.024) | - | 0.27596 | - |
| c.146A>G(p.Glu49Gly) | Damaging(0.001) | Disease causing | Deleterious(-4.03) | 24.4 | 0.789 | 0.3986 |
