# Supplementary material for: Outcomes of cochlear implants in patients with PCDH15 mutations: a clinical study
Source: Front Genet. 2025 May 22;16:1541333. doi: 10.3389/fgene.2025.1541333 (PMC12141853; doi:10.3389/fgene.2025.1541333)
Supplement: Supplementary file 2 [file Image2.pdf]

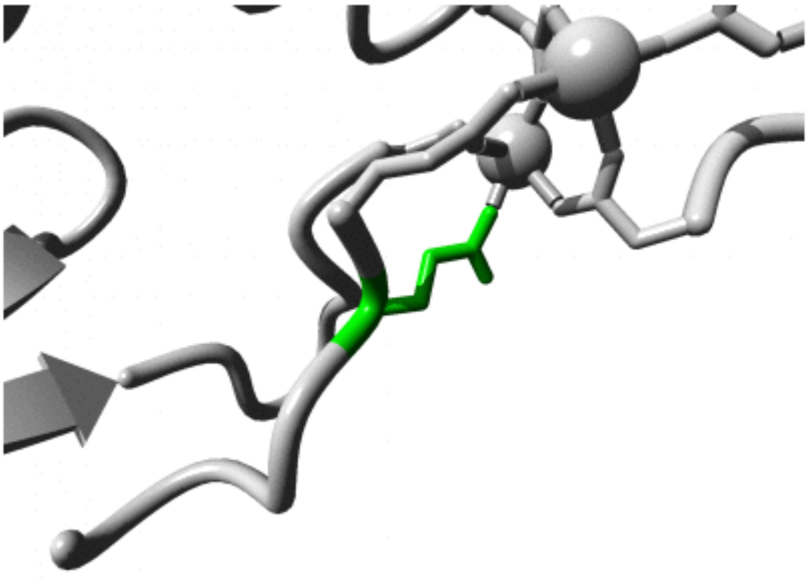

The wild-type side chain is shown in green

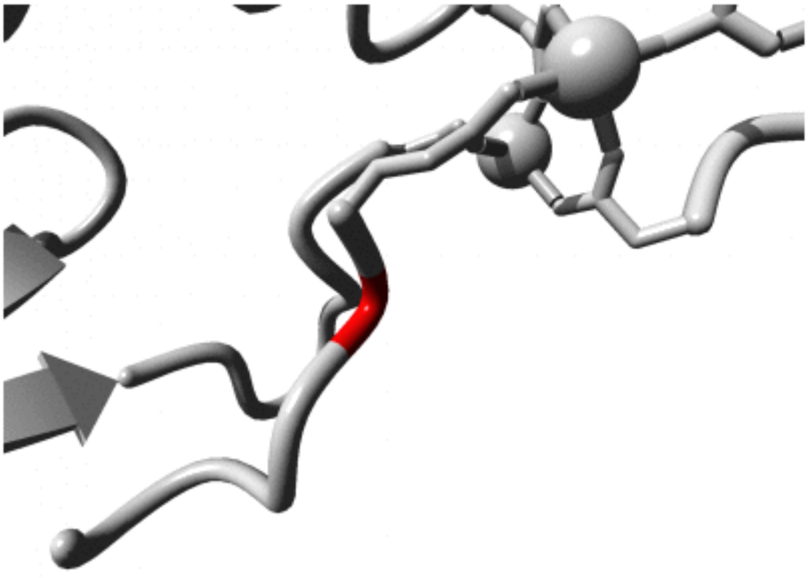

The mutant side chain is shown in red.

The HOPE online server (https://www3.cmbi.umcn.nl/hope/) predicted that the wild-type residue participates in metalion coordination (shown in green). Upon mutation, the loss of the negative charge in glutamic acid destabilizes the interaction with calcium (Ca) (shown in red), which may impair the stability of the protein's structural domain.
