# Supplementary material for: Outcomes of cochlear implants in patients with PCDH15 mutations: a clinical study
Source: Front Genet. 2025 May 22;16:1541333. doi: 10.3389/fgene.2025.1541333 (PMC12141853; doi:10.3389/fgene.2025.1541333)
Supplement: Supplementary file 3 [file Image1.pdf]

Family 1

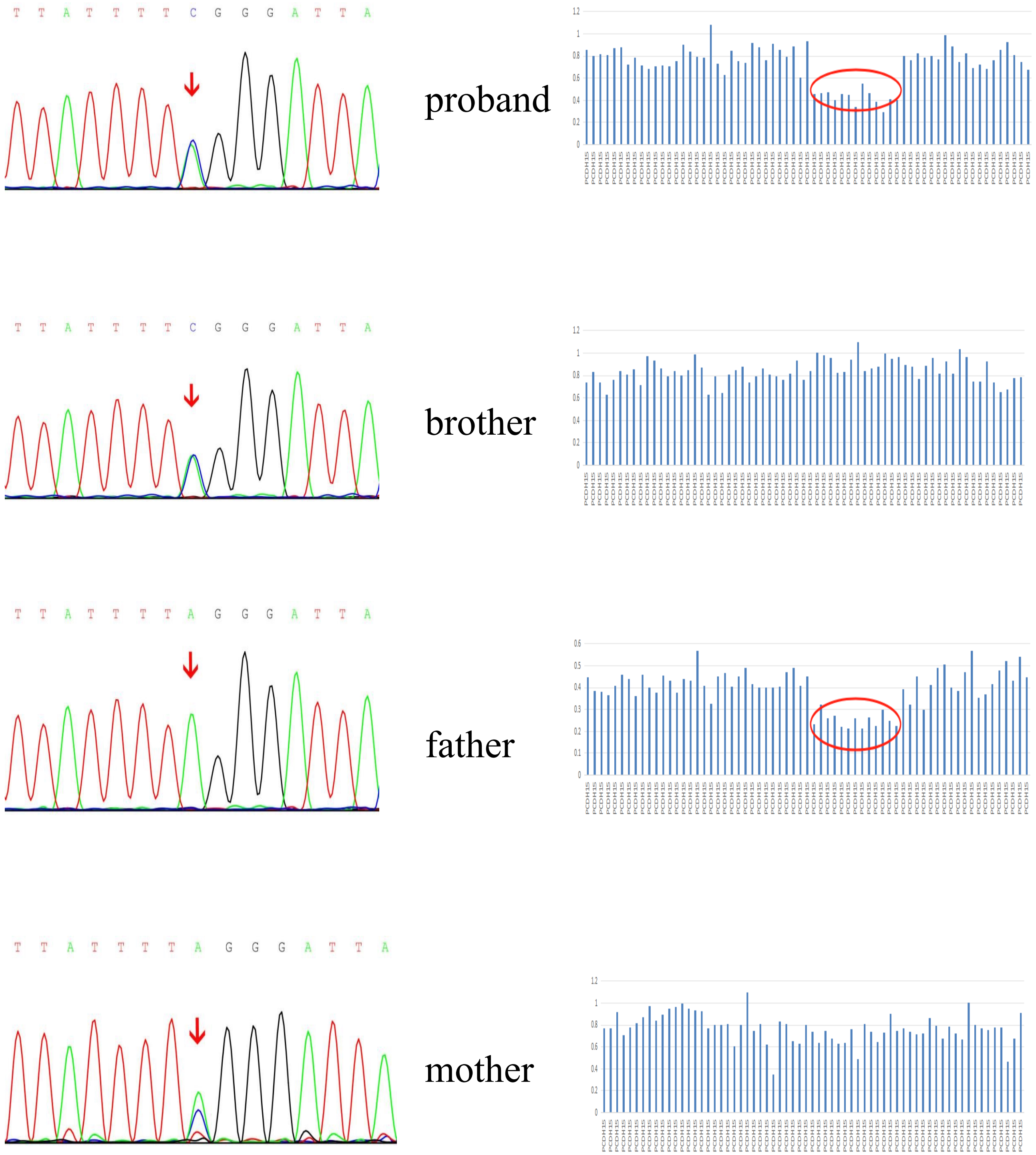

Family 1 pedigree: c.2869-2A>C locus validated in the first generation and results of exon 14-21 deletion.

Family 2 PCDH15 c.2367_2369del(p.Val790del)

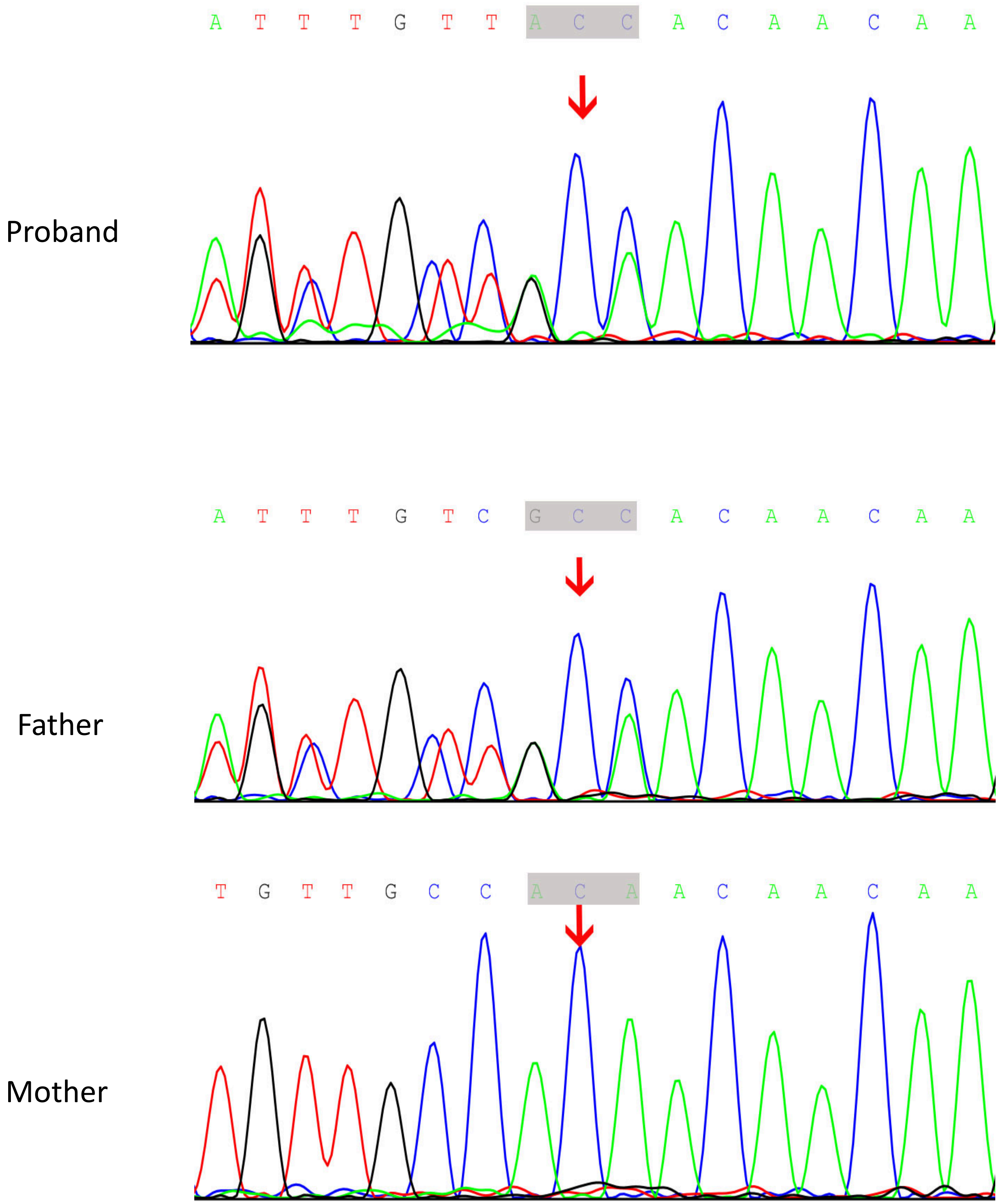

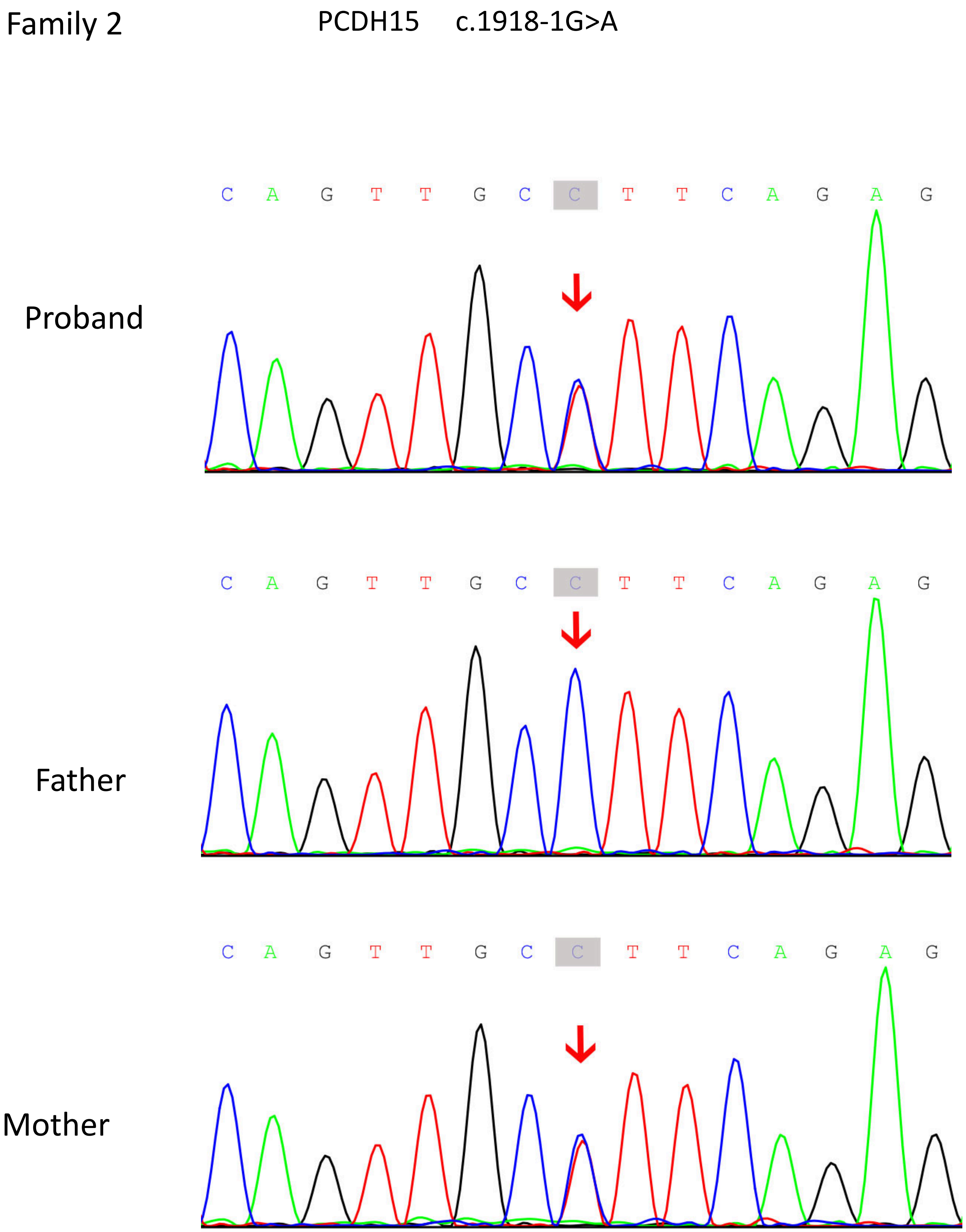
Family 2
PCDH15 c.1918-1G>A
Proband
C A G T T G C C T T C A G A G
Father
C A G T T G C C T T C A G A G
Mother
C A G T T G C C T T C A G A G

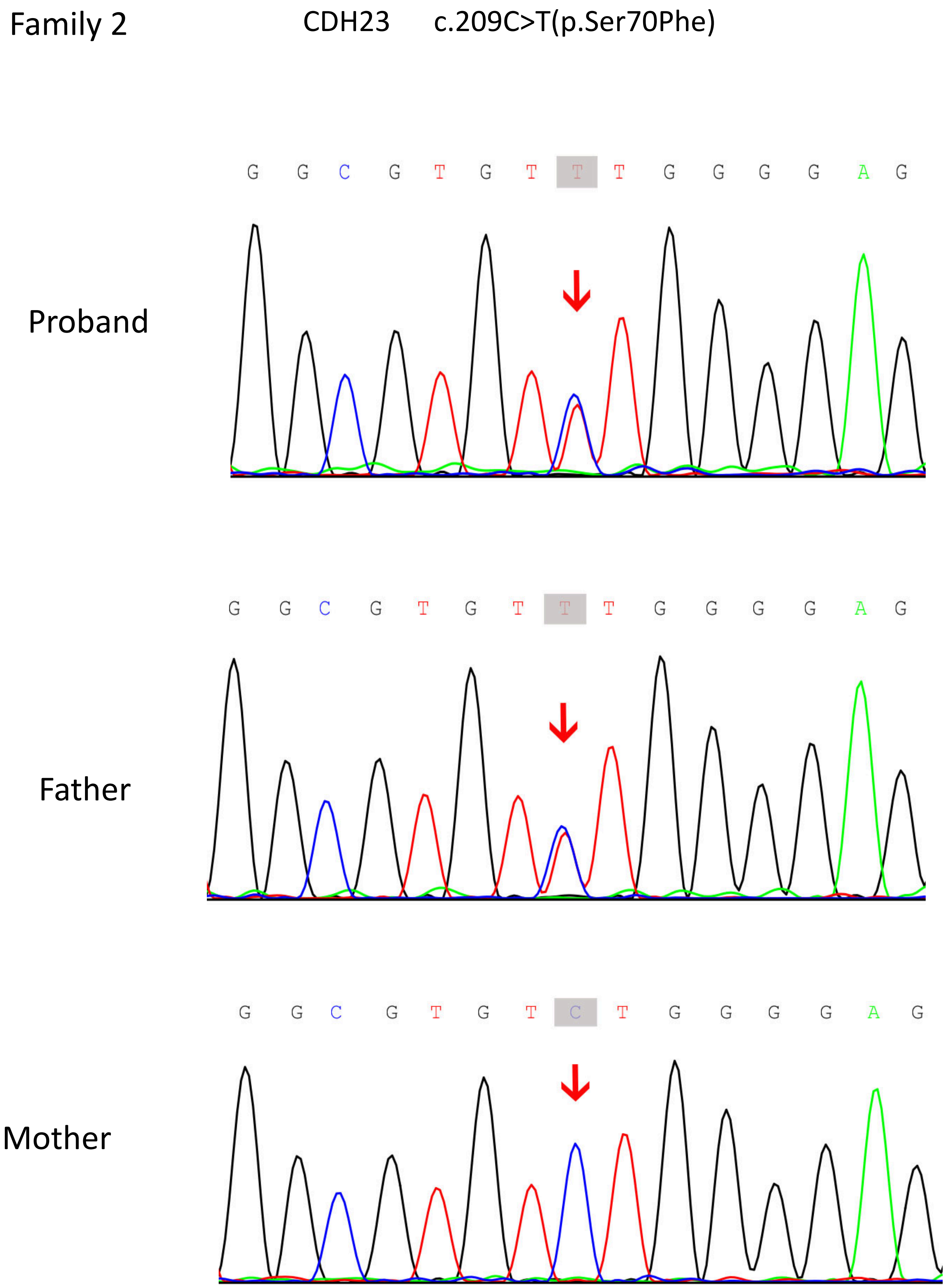
Family 2
CDH23 c.209C>T(p.Ser70Phe)
Proband
G G C G T G T T T G G G G A G
Father
G G C G T G T T T G G G G A G
Mother
G G C G T G T C T G G G G A G

Family 3

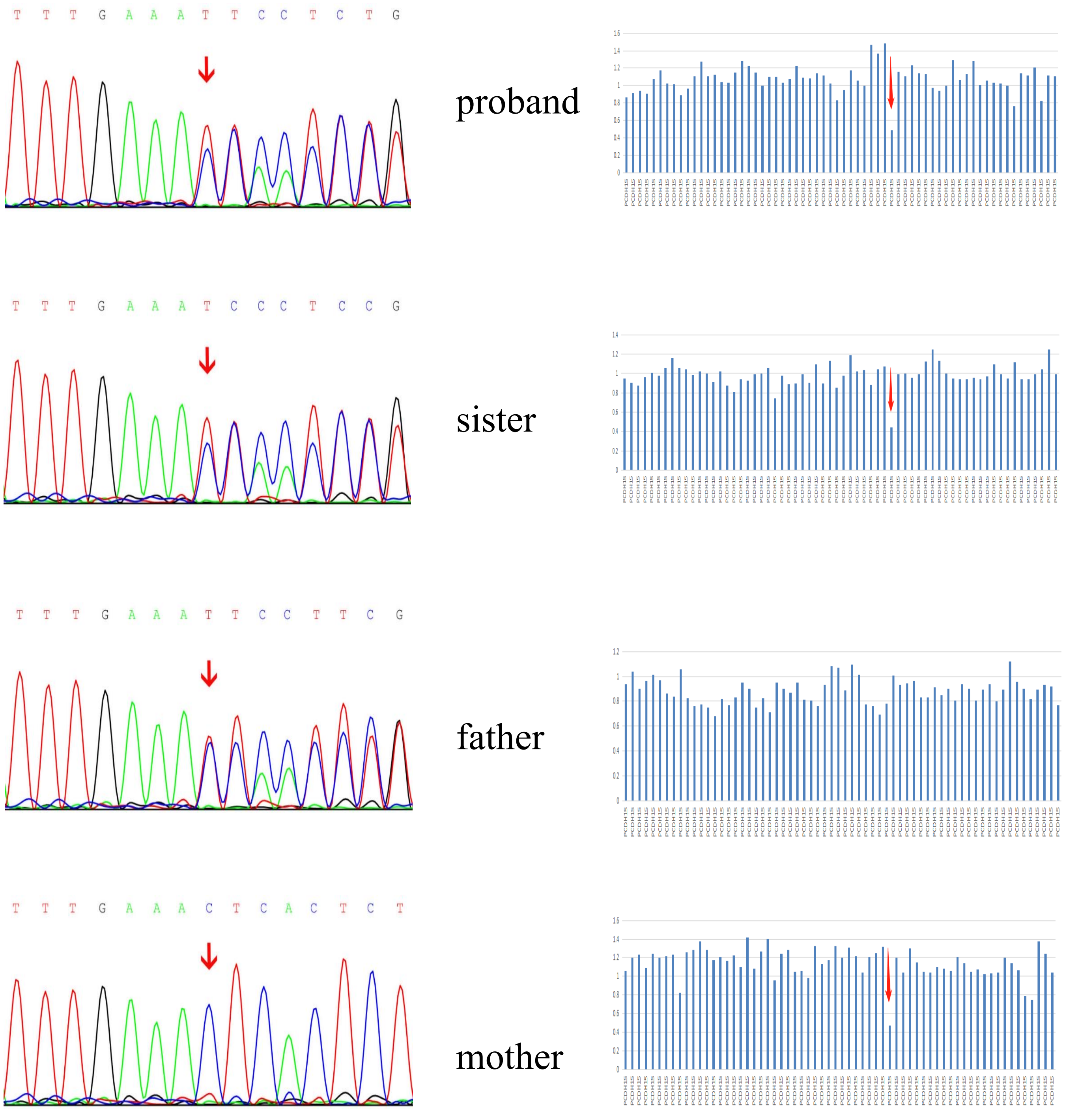

Family 3 c.4744del validated in the first generation and results of exon 19 deletion.

Family 4

proband

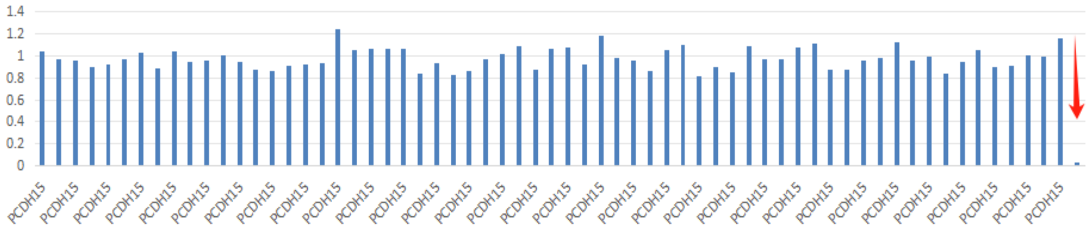

sister

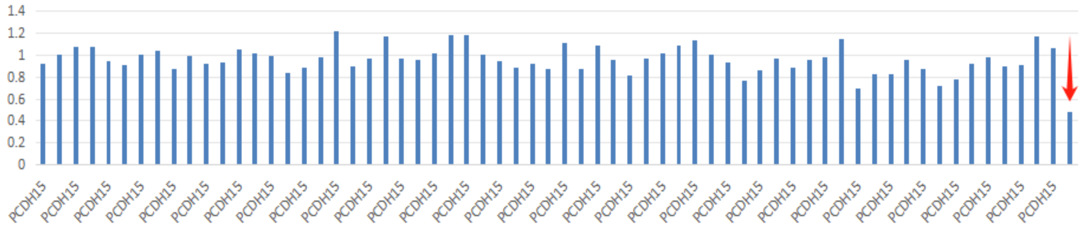

father

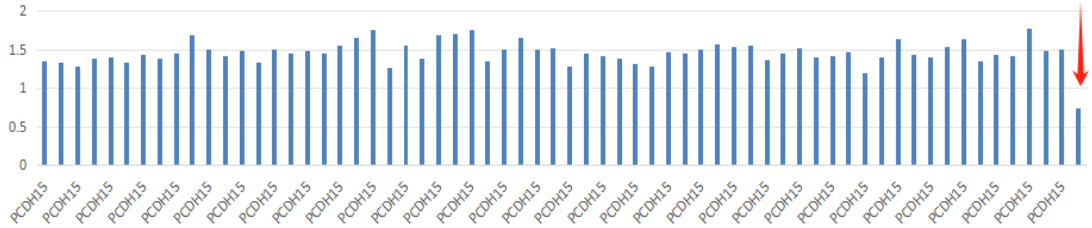

mother

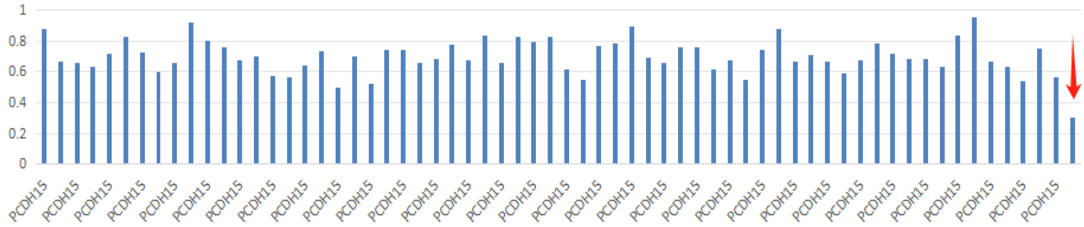

Family 4 Results of exon 2 deletion.

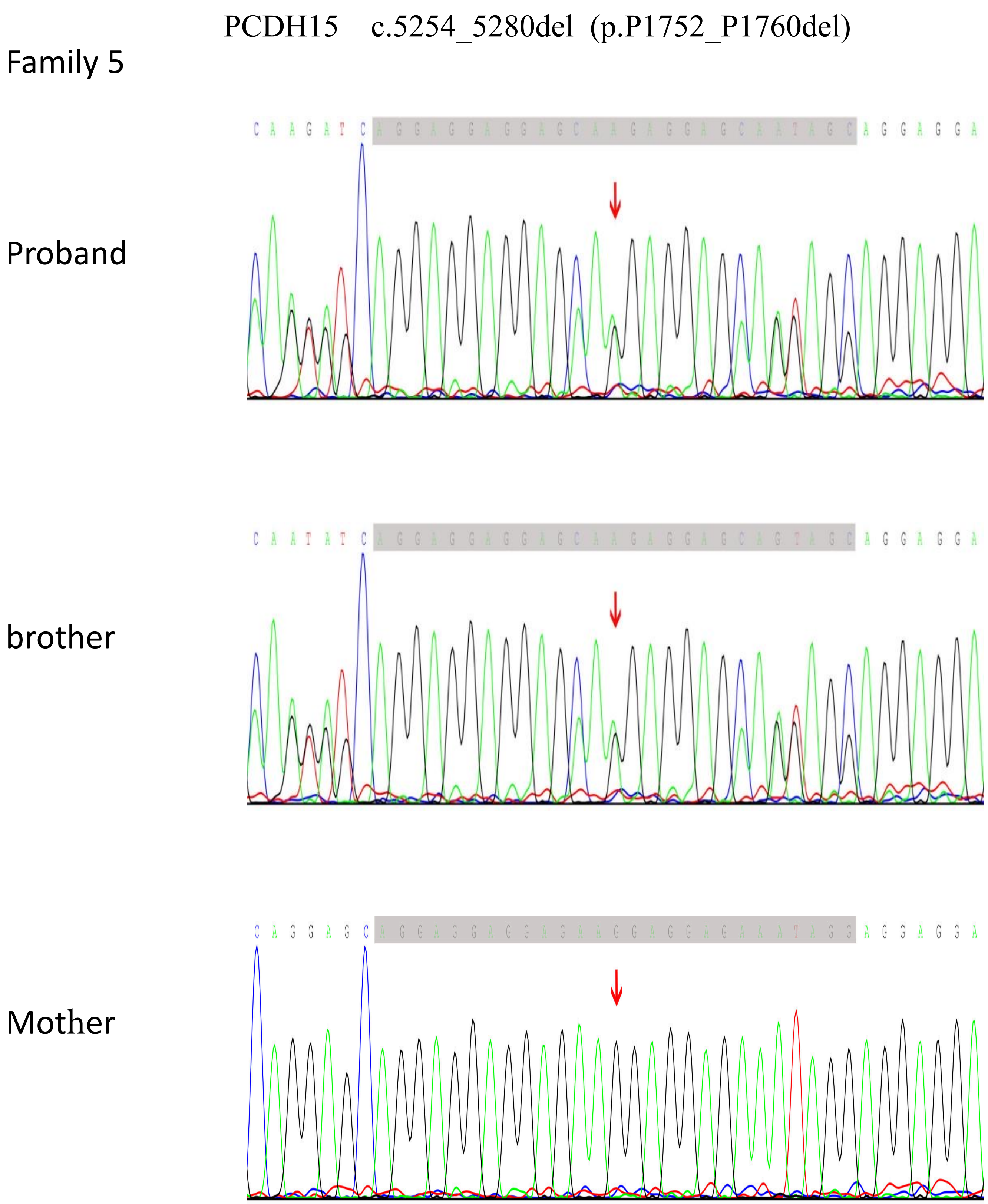
PCDH15 c.5254_5280del (p.P1752_P1760del)
Family 5
Proband
brother
Mother

Family 5

PCDH15 c.146A>G(p.Glu49Gly)

Proband

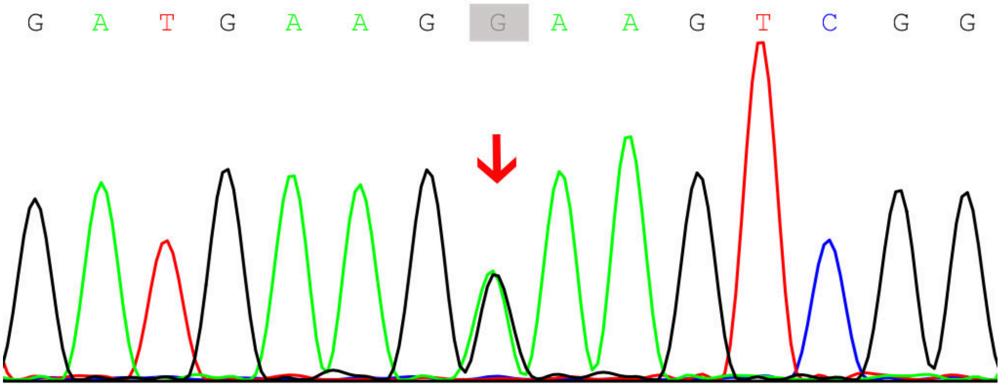

Brother

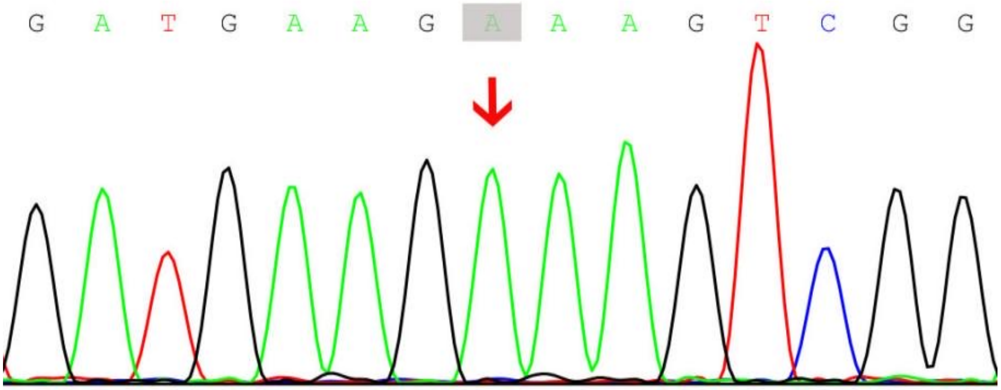

Mother

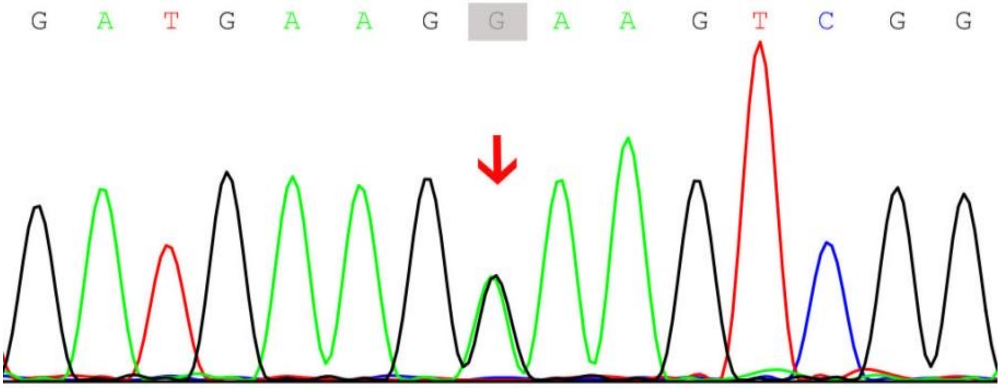

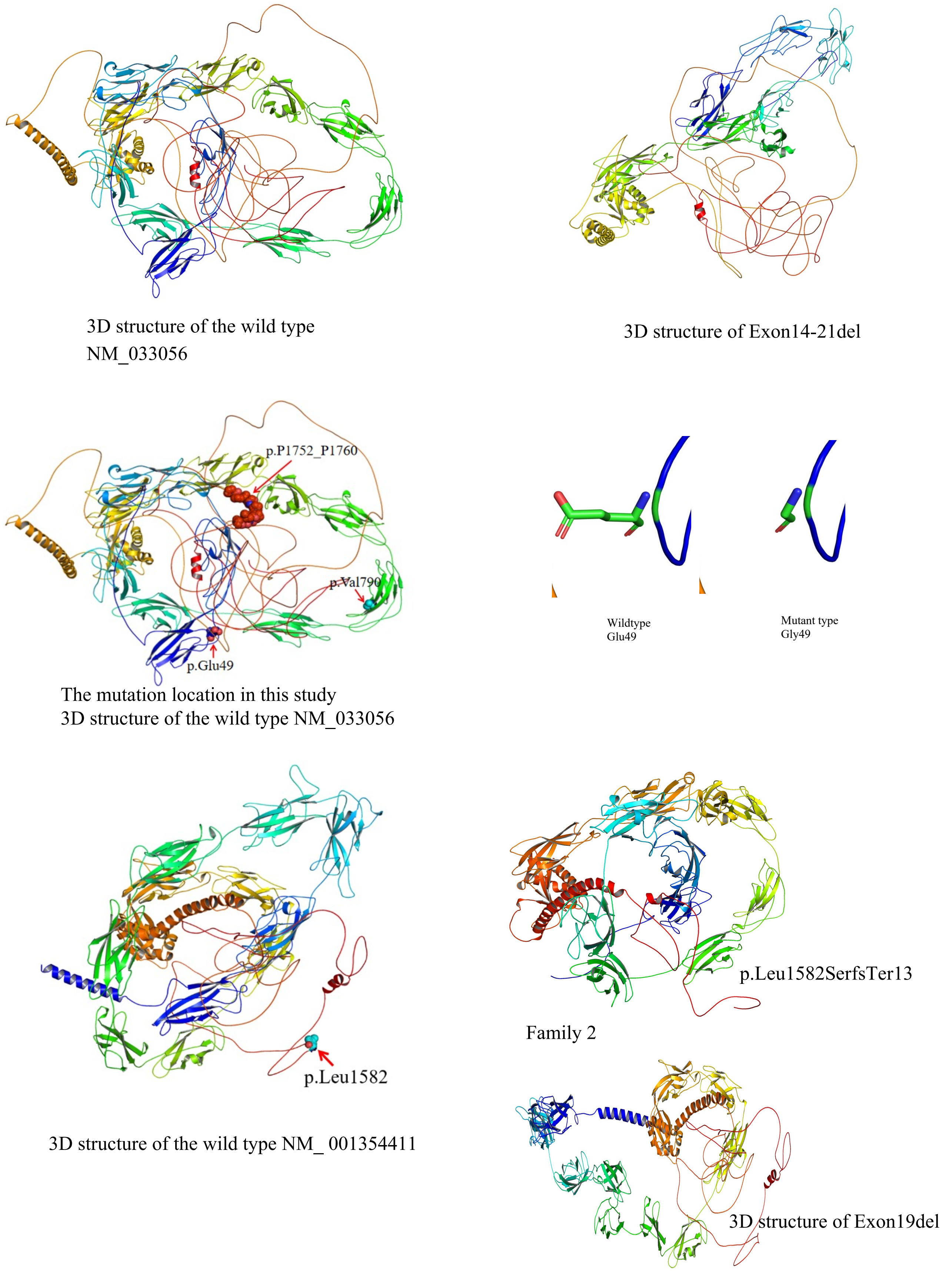

3D structural diagrams of wild-type and mutant PCDH15 proteins
